# Supplementary material for: Rapid, automated, and experimenter-free touchscreen testing reveals reciprocal interactions between cognitive flexibility and activity-based anorexia in female rats
Source: eLife. 2023 Jun 30;12:e84961. doi: 10.7554/eLife.84961 (PMC10344425; doi:10.7554/eLife.84961)
Supplement: Figure 2—source data 1. [file elife-84961-fig2-data1.docx]

**Figure 2 Statistics**

| **Figure** | **Statistical test** | **Group n** | **Main analysis result** | **Significant post-hoc multiple comparisons** |
| --- | --- | --- | --- | --- |
| **2A** | One-way RM ANOVA | n=8 | *F*(1.620, 11.34)= 4.249, ***p*=.0484** |  |
| **2B** | One-way RM ANOVA |  | *F*(1.465, 10.25)= 8.694, ***p*=.0092** | R1 > R2 ***p*=.0099**  R1 > R3 ***p*=.0070** |
| **2C** | One-way RM ANOVA |  | *F*(2.207, 15.45)= 7.994, ***p*=.0034** | R1 > R3 ***p*=.0035** |
| **2D** | Two-way RM ANOVA |  | Phase *F*(2.351, 49.37)=14.35, ***p*<.0001**  Outcome *F*(2, 21)=7.666, ***p*=.0032**  Interaction F(6, 63)=5.277, ***p*=.0002** | Incorrect: R1 > PD ***p*=.0014**; R1 > R3 ***p*=.0309**  Omission: PD > R3 ***p*=.0484**; R1 > R2 ***p*=.0092**; R1 > R3 ***p*=.0018** |
| **2E** | Two-way RM ANOVA |  | Phase *F*(2.351, 49.37)=14.35, ***p*<.0001**  Outcome *F*(2, 21)=7.666, ***p*=.0032**  Interaction F(6, 63)=5.277, ***p*=.0002** | R2: Correct > Omission ***p*=.0045**; Incorrect > Omission ***p*=.0059**  R3: Correct > Omission ***p*=.0005**; Incorrect > Omission ***p*=.0008** |
| **2G** | Mixed-effects analysis | Unlimited access n=10  Dark phase only n=10 | Stage *F*(2, 53)=4.151, ***p*=.0211**  Group *F*(1, 53)=9.103, ***p*=.0039**  Interaction *F*(2, 53)=1.156, *p*=.3226 | PD: Dark phase only > Unlimited access ***p*=.0371** |
| **2H** | Mixed-effect analysis |  | Stage *F*(2, 53)=10.74, ***p*=.0001**  Group *F*(1, 53)=9.663, ***p*=.0030**  Interaction *F*(2, 53)=2.249, *p*=.1155 | PD: Dark phase only > Unlimited access ***p*=.0030** |
| **2J** | Mixed-effect analysis |  | Stage *F*(2, 35)=1.374, *p*=.2663  Group *F*(1, 18)=3.607, *p*=.0737  Interaction *F*(2, 35)=.6190, *p*=.5443 |  |
| **2K** | Mixed-effect analysis |  | Stage *F*(2, 53)=.9793, *p*=.3823  Group *F*(1, 53)=12.79, ***p*=.0008**  Interaction *F*(2, 53)=3.176, ***p*=.0498** | PD: Unlimited access > Dark phase only ***p*=.0024**  R1: Unlimited access > Dark phase only ***p*=.0332** |

**Figure 2-figure supplement 2 Statistics**

| **Figure** | **Statistical test** | **Group n** | **Main analysis result** |
| --- | --- | --- | --- |
| **2S2A** | Two-way RM ANOVA | ABA Susceptible n=10  ABA  Resistant n=12 | ABA outcome *F*(1, 20)=0.223, *p*=.6421  Stage *F*(3, 60)=6.99, ***p*=.0004**  Interaction *F*(3, 60)=, *p*=.8570 |
| **2S2B** | Two-way RM ANOVA |  | ABA outcome *F*(1, 20)=0.172, *p*=.6825  Stage *F*(3, 60)=9.94, ***p*<.0001**  Interaction *F*(3, 60)=0.119, *p*=.9487 |
| **2S2C** | Two-way RM ANOVA |  | ABA outcome *F*(1, 20)=0.357, *p*=.6463  Stage *F*(3, 60)=1.66, *p*=.1851  Interaction *F*(3, 60)=0.490, *p*=.6903 |
| **2S2D** | Two-way RM ANOVA | ABA Naïve n=27  ABA Exposed n=22 | ABA Exposure *F*(1, 47)=0.467, *p*=.4977  Stage *F*(3, 141)=15.5, ***p*<.0001**  Interaction *F*(3, 141)=1.55, *p*=.2032 |
| **2S2E** | Two-way RM ANOVA |  | ABA Exposure *F*(1, 47)=0.233, *p*=.6313  Stage *F*(3, 141)=23.8, ***p*<.0001**  Interaction *F*(3, 141)=1.51, *p*=.2152 |
| **2S2F** | Two-way RM ANOVA |  | ABA Exposure *F*(1, 47)=0.0456, *p*=.8319  Stage *F*(3, 141)=3.59, ***p*=.0154**  Interaction *F*(3, 141)=0.925, *p*=.4306 |
| **2S2G** | Two-way RM ANOVA | ABA Exposed + Learned task n=11  ABA Exposed + Did not learn n=11 | R1 outcome *F*(1, 20)=0.00, *p*>.9999  Stage *F*(3, 60)=7.77, ***p*=.0002**  Interaction *F*(3, 60)=0.206, *p*=.8917 |
| **2S2H** | Two-way RM ANOVA |  | R1 outcome *F*(1, 20)=0.0833, *p*=.7758  Stage *F*(3, 60)=11.4, ***p*<.0001**  Interaction *F*(3, 60)=0.00218, *p*=.9999 |
| **2S2I** | Two-way RM ANOVA |  | R1 outcome *F*(1, 20)=0.143, *p*=.7092  Stage *F*(3, 60)=2.97, ***p*=.0388**  Interaction *F*(3, 60)=0.425, *p*=.7357 |
