# Supplementary material for: Rapid, automated, and experimenter-free touchscreen testing reveals reciprocal interactions between cognitive flexibility and activity-based anorexia in female rats
Source: eLife. 2023 Jun 30;12:e84961. doi: 10.7554/eLife.84961 (PMC10344425; doi:10.7554/eLife.84961)
Supplement: Figure 3—source data 1. [file elife-84961-fig3-data1.docx]

**Figure 3 Statistics**

| **Figure** | **Statistical test** | **Group n** | **Main analysis result** | **Significant post-hoc multiple comparisons** |
| --- | --- | --- | --- | --- |
| **3B** | Two-way RM ANOVA  Only includes animals that learned the task | ABA Susceptible n=10  ABA Resistant  n=12 | Stage *F*(1, 20)=62.1, ***p*<.0001**  ABA outcome *F*(1, 20)=3.39, *p*=.0806  Interaction *F*(1, 20)=5.52, ***p*=.0292** | R1: ABA Resistant > ABA Susceptible ***p*=.0142** |
| **3C** | Two-way RM ANOVA  Only includes animals that learned the task |  | Stage *F*(1, 20)=84.9, ***p*<.0001**  ABA outcome *F*(1, 20)=1.53, *p*=.2302  Interaction *F*(1, 20)=2.98, *p*=.0998 |  |
| **3D** | Two-way RM ANOVA  Only includes animals that learned the task |  | Trial outcome *F*(1, 20)=33.3, ***p*<.0001**  ABA outcome *F*(1, 20)=0.0523, *p*=.8214  Interaction *F*(1, 20)=0.188, *p*=.6692 |  |
| **3E** | Two-way RM ANOVA  Only includes animals that learned the task |  | Trial outcome *F*(1, 20)=38.9, ***p*<.0001**  ABA outcome *F*(1, 20)=2.35, *p*=.1407  Interaction *F*(1, 20)=4.88, ***p*=.0389** | Non-correct trials: ABA Resistant > ABA Susceptible ***p*=.0401** |

**Figure 3-figure supplement 1 Statistics**

| **Figure** | **Statistical test** | **Group n** | **Main analysis result** | **Significant post-hoc multiple comparisons** |
| --- | --- | --- | --- | --- |
| **3S1B** | Unpaired t test | ABA Susceptible n=11  ABA Resistant n=14 | *t*(23)=11.45, ***p*<.0001** |  |
| **3S1C** | Unpaired t test |  | *t*(23)=7.799, ***p*<.0001** |  |
| **3S1D** | Baseline: Mixed-effects analysis | ABA Susceptible n=10  ABA Resistant n=13 | Time *F*(6, 113)=23.1, ***p*<.0001**  ABA outcome *F*(1, 21)=0.122, *p*=.7301  Interaction *F*(6, 113)=0.729, *p*=.6275 |  |
| **3S1E** | Two-way RM ANOVA |  | Phase *F*(1, 21)=219.8, ***p*<.0001**  ABA outcome *F*(1, 21)=1.573, *p*=.2235  Interaction *F*(1, 21)=5.992, ***p*=.0232** | ABA: ABA Susceptible > ABA Resistant ***p*=.0497** |
| **3S1F** | Unpaired t test |  | *t*(21)=2.448, ***p*=.0232** |  |
| **3S1G** | Two-way RM ANOVA |  | Phase *F*(1, 21)=17.79, ***p*=.0004**  ABA outcome *F*(1, 21)=24.94, ***p*<.0001**  Interaction *F*(1, 21)=0.9967, *p*=.3295 | Baseline: ABA Resistant > ABA Susceptible ***p*=.0011**  ABA: ABA Resistant > ABA Susceptible ***p*<.0001** |
| **3S1H** | Two-way ANOVA | PD: ABA Susceptible n=7 (55 videos)  ABA Resistant n=9 (35 videos)  R1: ABA Susceptible n=10 (119 videos)  ABA Resistant n=11 (107 videos) | Stage *F*(1, 160)=0.0171, *p*=.8960  ABA outcome *F*(1, 160)=0.146, *p*=.7033  Interaction *F*(1, 160)=0.0168, *p*=.8971 |  |
| **3S1I** | Two-way ANOVA |  | Stage *F*(1, 160)=15.6, ***p*=.0001**  ABA outcome *F*(1, 160)=0.758, *p*=.3851  Interaction *F*(1, 160)=2.57, *p*=.1110 | R1: ABA Susceptible > ABA Resistant ***p*=.0101** |
| **3S1J** | Two-way ANOVA |  | Stage *F*(1, 160)=5.96, ***p*=.0157**  ABA outcome *F*(1, 160)=0.229, *p*=.6332  Interaction *F*(1, 160)=1.01, *p*=.3171 |  |
| **3S1K** | Two-way ANOVA |  | Stage *F*(1, 160)=1.01, *p*=.3174  ABA outcome *F*(1, 160)=0.909, *p*=.3418  Interaction *F*(1, 160)=0.142, *p*=.7067 |  |
| **3S1L** | Two-way ANOVA |  | Stage *F*(1, 160)=0.00711, *p*=.9329  ABA outcome *F*(1, 160)=0.0799, *p*=.7777  Interaction *F*(1, 160)=0.122, *p*=.7274 |  |
| **3S1M** | Two-way ANOVA |  | Stage *F*(1, 160)=0.464, *p*=.4969  ABA outcome *F*(1, 160)=0.0155, *p*=.9012  Interaction *F*(1, 160)=0.423, *p*=.5163 |  |
| **3S1N** | Two-way ANOVA |  | Stage *F*(1, 160)=0.690, *p*=.4074  ABA outcome *F*(1, 160)=0.191, *p*=.6628  Interaction *F*(1, 160)=0.657, *p*=.4188 |  |

**Figure 3 -figure supplement 3 Statistics**

| **Figure** | **Statistical test** | **Group n** | **Main analysis result** | **Significant post-hoc multiple comparisons** |
| --- | --- | --- | --- | --- |
| **3S3A** | Two-way ANOVA  Only includes animals that learned the task | ABA Susceptible  n=7 (55 videos)  ABA Resistant n=9 (35 videos) | Behaviour *F*(5, 528)=268.5, ***p*<.0001**  ABA outcome *F*(1, 528)=8.286e-012, *p*>.9999  Interaction *F*(5, 528)=12.21, ***p*<.0001** | Inactive: ABA Susceptible > ABA Resistant ***p*<.0001**  Locomote: ABA Resistant > ABA Susceptible ***p*=.0190**  Rearing: ABA Resistant > ABA Susceptible ***p*=.0336** |
| **3S3B** | Two-way ANOVA  Only includes animals that learned the task | ABA Susceptible n=10 (119 videos)  ABA Resistant n=11 (107 videos) | Behaviour *F*(5, 1344)=723.6, ***p*<.0001**  ABA outcome *F*(1, 1344)=4.623e-012, *p*>.9999  Interaction *F*(5, 1344)=12.82, ***p*<.0001** | Inactive: ABA Susceptible > ABA Resistant ***p*<.0001**  Rearing: ABA Resistant > ABA Susceptible ***p*=.0384** |
