# Supplementary material for: Rapid, automated, and experimenter-free touchscreen testing reveals reciprocal interactions between cognitive flexibility and activity-based anorexia in female rats
Source: eLife. 2023 Jun 30;12:e84961. doi: 10.7554/eLife.84961 (PMC10344425; doi:10.7554/eLife.84961)
Supplement: Figure 4—source data 1. [file elife-84961-fig4-data1.docx]

**Figure 4 Statistics**

| **Figure** | **Statistical test** | **Group n** | **Main analysis result** | **Significant post-hoc multiple comparisons** |
| --- | --- | --- | --- | --- |
| **4C** | Two-way RM ANOVA  Only includes animals that learned the task | ABA Naïve n=25  ABA Exposed  n=11  Food Restriction Only  n=15 | Stage *F*(1, 48)=146, ***p*<.0001**  ABA exposure *F*(2, 48)=5.55, ***p*=.0068**  Interaction *F*(2,48)=0.491, *p*=.6152 | ABA Exposed > ABA Naïve ***p*=.0051**  PD: ABA Exposed + Learned task > ABA Naïve + Learned task ***p*=.0098**  R1: ABA Exposed + Learned task > ABA Naïve + Learned task ***p*=.0205** |
| **4D** | Two-way RM ANOVA  Only includes animals that learned the task |  | Stage *F*(1, 48)=182, ***p*<.0001**  ABA exposure *F*(2,48)=2.18, *p*=.1240  Interaction *F*(2,48)=0.348, *p*=.7078 |  |
| **4E** | Two-way RM ANOVA  Only includes animals that learned the task |  | Outcome *F*(2, 96)=244, ***p*<.0001**  ABA exposure *F*(2, 48)=4.37, ***p*=.0181**  Interaction *F*(4, 96)=1.41, *p*=.2379 | ABA Exposed > ABA Naïve ***p*=.0241**  ABA Exposed > Food restriction only ***p*=.0412**  Correct: ABA Exposed + Learned task > ABA Naïve + Learned task ***p*=.0185**; ABA Exposed + Learned task > Food restriction only + Learned task ***p*=.0259**  Incorrect: ABA Exposed + Learned task > Food restriction only + Learned task ***p*=.0479**  Omission: ABA Exposed + Learned task > ABA Naïve + Learned task ***p*=.0224** |
| **4F** | Two-way RM ANOVA  Only includes animals that learned the task |  | Outcome *F*(2, 144)=31.73, ***p*<.0001**  ABA exposure *F*(2, 144)=1.338, *p*=.2656  Interaction *F*(4, 144)=0.5782 , *p*=.6789 |  |
| **4G** | Unpaired t test | ABA Exposed + Learned task n=11  ABA Exposed + Did not learn n=11 | *t*(20)=0.1987, *p*=.8445 |  |
| **4H** | Unpaired t test |  | *t*(20)=0.5888, *p*=.5626 |  |
| **4I** | Two-way RM ANOVA |  | Trial outcome *F*(1.706, 34.12)=71.94, ***p*<.0001**  Learning outcome *F*(1, 20)=0.7749, *p*=.3892  Interaction *F*(2, 40)=0.3061, *p*=.7380 |  |

**Figure 4-figure supplement 1 Statistics**

| **Figure** | **Statistical test** | **Group n** | **Main analysis result** | **Significant post-hoc multiple comparisons** |
| --- | --- | --- | --- | --- |
| **4S1J** | Unpaired t test | Food restriction only + Learned task n=15  Food restriction only + Did not learn n=7 | *t*(20)=0.7458, *p*=.4645 |  |
| **4S1K** | Unpaired t test |  | *t*(20)=5134, *p*=.6133 |  |
| **4S1L** | Two-way RM ANOVA |  | Trial outcome *F*(1.628, 32.55)=43.09, ***p*<.0001**  Learning outcome *F*(1, 20)=0.2636, *p*=.6133  Interaction *F*(2, 40)=2.573, *p*=.0888 |  |
| **4S1M** | Two-way RM ANOVA | ABA Naïve n=28 (Learned task n=25, Did not learn n=3)  ABA Exposed + Learned task n=11  ABA Exposed + Did not learn n=11  Food restriction only + Learned task n=15  Food restriction only + Did not learn n=7 | Stage *F*(1, 67)=150.0, ***p*<.0001**  Group *F*(4, 67)=12.0, ***p*<.0001**  Interaction *F*(4, 67)=10.8, ***p*<.0001** | R1: ABA Naïve > ABA Exposed + Did not learn ***p*<.0001**; ABA Naïve > Food restriction only + Did not learn ***p*<.0001**; ABA Exposed + Learned task > ABA Exposed + Did not learn ***p*<.0001**; ABA Exposed + Learned task > Food restriction only+ Did not learn ***p*<.0001**; Food restriction only + Learned task > ABA Exposed + Did not learn ***p*<.0001**; Food restriction only + Learned task > Food restriction only + Did not learn ***p*<.0001** |
| **4S1N** | Two-way RM ANOVA |  | Stage *F*(1, 67)=12.4, ***p*=.0008**  Group *F*(4, 67)=10.3, ***p*<.0001**  Interaction *F*(4, 67)=7.94, ***p*<.0001** | R1: ABA Naïve > ABA Exposed + Did not learn ***p*<.0001**; ABA Naïve > Food restriction only + Did not learn ***p*<.0001**; ABA Exposed + Learned task > ABA Exposed + Did not learn ***p=*.0003**; ABA Exposed + Learned task > Food restriction only+ Did not learn ***p*=.0002**; Food restriction only + Learned task > ABA Exposed + Did not learn ***p*<.0001**; Food restriction only + Learned task > Food restriction only + Did not learn ***p*<.0001** |
| **4S1O** | Two-way RM ANOVA |  | Stage *F*(1, 67)=20.0, ***p*<.0001**  Group *F*(4, 67)=1.97, *p*=.1095  Interaction *F*(4, 67)=0.614, *p*=.6538 |  |

**Figure 4-figure supplement 3 Statistics**

| **Figure** | **Statistical test** | **Group n** | **Main analysis result** | **Significant post-hoc multiple comparisons** |
| --- | --- | --- | --- | --- |
| **4S3A** | Two-way ANOVA | PD: ABA Naïve n=12 (95 videos)  ABA Exposed n=6 (49 videos)  R1: ABA Naïve n=12 (275 videos)  ABA Exposed n=6 (88 videos) | Behaviour *F*(5, 852)=402.8, ***p*<.0001**  ABA timing *F*(1, 852)=2.058e-012, *p*>.9999  Interaction *F*(5, 852)=15.23, ***p*<.0001** | Inactive: ABA Exposed > ABA Naïve ***p*<.0001**  Investigating: ABA Naïve > ABA Exposed ***p*=.0078**  Rotate Body: ABA Naïve > ABA Exposed ***p*=.0002** |
| **4S3B** | Two-way ANOVA |  | Behaviour *F*(5, 2166)=987.0, ***p*<.0001**  ABA timing *F*(1, 2166)=2.443e-011, *p*>.9999  Interaction *F*(5, 2166)=3.629, ***p*=.0028** | Rotate Body: ABA Naïve > ABA Exposed ***p*=.0039** |
| **4S3C** | Two-way ANOVA |  | Stage *F*(1, 476)=6.116, ***p*=.0137**  ABA timing *F*(1, 476)=44.62, ***p*<.0001**  Interaction *F*(1, 476)=0.02162, *p*=.8832 | PD: ABA Exposed > ABA Naïve ***p*=.0001**  R1: ABA Exposed > ABA Naïve ***p*<.0001** |
| **4S3D** | Two-way ANOVA |  | Stage *F*(1 ,476)=26.11, ***p*<.0001**  ABA timing *F*(1, 476)=4.149, ***p*=.0422**  Interaction *F*(1, 476)=0.07958, *p*=.7780 |  |
| **4S3E** | Two-way ANOVA |  | Stage *F*(1, 476)=30.64, ***p*<.0001**  ABA timing *F*(1, 476)=10.34, ***p*=.0014**  Interaction *F*(1, 476)=0.6610, *p*=.4166 | PD: ABA Naïve > ABA Exposed ***p*=.0277** |
| **4S3F** | Two-way ANOVA |  | Stage *F*(1 ,476)=21.24, ***p*<.0001**  ABA timing *F*(1, 476)=7.833, ***p*=.0053**  Interaction *F*(1, 476)=0.8649, *p*=.3528 | PD: ABA Naïve > ABA Exposed ***p*=.0454** |
| **4S3G** | Two-way ANOVA |  | Stage *F*(1, 476)=29.17, ***p*<.0001**  ABA timing *F*(1, 476)=15.49, ***p*<.0001**  Interaction *F*(1, 476)=2.615, *p*=.1066 | PD: ABA Naïve > ABA Exposed ***p*=.0014** |
| **4S3H** | Two-way ANOVA |  | Stage *F*(1, 476)=15.78, ***p*<.0001**  ABA timing *F*(1, 476)=2.965, ***p*=.0857**  Interaction *F*(1, 476)=0.4845, *p*=.4867 |  |
| **4S3I** | Two-way ANOVA |  | Stage *F*(1, 476)=0.1234, *p*=.7256  ABA timing *F*(1, 476)=1.185, *p*=.2769  Interaction *F*(1, 476)=0.003303, *p*=.9542 |  |

**Figure 4-figure supplement 4 Statistics**

| **Figure** | **Statistical test** |  | **Main analysis result** | **Significant post-hoc multiple comparisons** |
| --- | --- | --- | --- | --- |
| **4S4A** | Two-way ANOVA | PD: Learners n=3 (21 videos)  Non-learners n=3 (28 videos)  R1: Learners n=3 (31 videos)  Non-learners n=4 (57 videos) | Behaviour *F*(5, 282)=158.9, ***p*<.0001**  Learning outcome *F*(1, 282)=3.255e-012, *p*>.9999  Interaction *F*(5, 282)=3.481, ***p*=.0045** | Inactive: Non-learners > Learners ***p*=.0059** |
| **4S4B** | Two-way ANOVA |  | Behaviour *F*(5, 516)=377.3, ***p*<.0001**  Learning outcome *F*(1, 516)=5.790e-013, *p*>.9999  Interaction *F*(5, 516)=13.08, ***p*<.0001** | Inactive: Non-learners > Learners ***p*<.0001**  Investigating: Learners > Non-learners ***p*=.0006** |
| **4S4C** | Two-way ANOVA |  | Stage *F*(1, 129)=3.167, *p*=.0775  Learning outcome *F*(1, 129)=0.3736, *p*=.5421  Interaction *F*(1, 129)=0.9221, *p*=.3387 |  |
| **4S4D** | Two-way ANOVA |  | Stage *F*(1, 129)=9.561, ***p*=.0024**  Learning outcome *F*(1, 129)=9.086, ***p*=.0031**  Interaction *F*(1, 129)=0.2754, *p*=.6006 | R1: ABA Exposed learned > did not learn ***p*=.0100** |
| **4S4E** | Two-way ANOVA |  | Stage *F*(1, 129)=9.531, ***p*=.0025**  Learning outcome *F*(1, 129)=3.115, *p*=.0800  Interaction *F*(1, 129)=5.691, ***p*=.0185** | R1: ABA Exposed learned > did not learn ***p*=.0021** |
| **4S4F** | Two-way ANOVA |  | Stage *F*(1, 129)=3.077, ***p*=.0818**  Learning outcome *F*(1, 129)=3.728, *p*=.0557  Interaction *F*(1, 129)=1.717, *p*=.1924 | R1: ABA Exposed learned > did not learn ***p*=.0199** |
| **4S4G** | Two-way ANOVA |  | Stage *F*(1, 129)=5.302, ***p*=.0229**  Learning outcome *F*(1, 129)=4.105, ***p*=.0448**  Interaction *F*(1, 129)=1.637, *p*=.2030 | R1: ABA Exposed learned > did not learn ***p*=.0172** |
| **4S4H** | Two-way ANOVA |  | Stage *F*(1, 129)=2.182, *p*=.1420  Learning outcome *F*(1, 129)=1.607, *p*=.2072  Interaction *F*(1, 129)=9.539, ***p*=.0025** | R1: ABA Exposed learned > did not learn ***p*=.0012** |
| **4S4I** | Two-way ANOVA |  | Stage *F*(1, 129)=0.02659, *p*=.8707  Learning outcome *F*(1, 129)=6.264, ***p*=.0136**  Interaction *F*(1, 129)=1.332, *p*=.2506 | R1: ABA Exposed learned > did not learn ***p*=.0075** |

**Figure 4-figure supplement 5 Statistics**

| **Figure** | **Statistical test** | **Group n** | **Main analysis result** | **Significant post-hoc multiple comparisons** |
| --- | --- | --- | --- | --- |
| **4S5A1** | Two-way ANOVA for each of Correct, Incorrect, Omission and Percent correct | ABA Susceptible n=12  ABA Resistant n=13 | Correct  ABA outcome *F*(1, 23)=0.3483, *p*=.5608  Session *F*(2.081, 47.86)=254.4, ***p*<.0001**  Interaction *F*(3 69,)=0.1649, *p*=.9197  Incorrect  ABA outcome *F*(1, 23)=0.4796, *p*=.4955  Session *F*(1.810, 41.63)=65.84, ***p*<.0001**  Interaction *F*(3, 69)=0.1807, *p*=.9092  Omission  ABA outcome *F*(1, 23)=0.8288, *p*=.3721  Session *F*(1.715, 39.44)=17.59, ***p*<.0001**  Interaction *F*(3, 69)=1.111, *p*=.3505  Percent correct  ABA outcome *F*(1, 23)=0.04614, *p*=.8318  Session *F*(1.994, 45.86)=361.4, ***p*<.0001**  Interaction *F*(3, 69)=0.07268, *p*=.9744 |  |
| **4S5A2** | Two-way ANOVA for each of Correct, Incorrect, Omission and Percent correct |  | Correct  ABA outcome *F*(1, 23)=4.338, ***p*=.0486**  Session *F*(1.332, 30.64)=133.2, ***p*<.0001**  Interaction *F*(3, 69)=1.972, *p*=1262  Incorrect  ABA outcome *F*(1, 23)=2.082, *p*=.1626  Session *F*(1.400, 32.20)=65.91, ***p*<.0001**  Interaction *F*(3, 69)=0.5522, *p*=.6484  Omission  ABA outcome *F*(1, 23)=0.3790, *p*=.5442  Session *F*(1.712, 39.38)=28.32, ***p*<.0001**  Interaction *F*(3, 69)=1.222, *p*=.3085  Percent correct  ABA outcome *F*(1, 23)=2.493, *p*=.1280  Session *F*(1.640, 37.72)=497.4, ***p*<.0001**  Interaction *F*(3, 69)=1.726, *p*=.1677 |  |
| **4S5B1** | Two-way ANOVA for each of Correct, Incorrect, Omission and Percent correct | ABA Exposed + Learned task n=11  ABA Exposed + Did not learn n=11 | Correct  Learning outcome *F*(1, 20)=90.92, ***p*<.0001**  Session *F*(1.806, 36.12)=293.5, ***p*<.0001**  Interaction *F*(3, 60)=57.94, ***p*<.0001**  Incorrect  Learning outcome *F*(1, 20)=0.01847, *p*=.8932  Session *F*(2.085, 41.70)=22.95, ***p*<.0001**  Interaction *F*(3, 60)=2.084, *p*=.1118  Omission  Learning outcome *F*(1, 20)=6.487, ***p*=.0192**  Session *F*(2.289, 45.77)=14.51, ***p*<.0001**  Interaction *F*(3, 60)=6.111, ***p*=.0011**  Percent correct  Learning outcome *F*(1, 20)=30.51, ***p*<.0001**  Session *F*(2.152, 43.03)=265.3, ***p*<.0001**  Interaction *F*(3, 60)=47.51, ***p*<.0001** | Correct  Last R1: Learned > Did not learn ***p*<.0001**  Omission  Last R1: Learned < Did not learn ***p*=.0120**  Percent correct  Last R1: Learned > Did not learn ***p*<.0001** |
| **4S5B2** | Two-way ANOVA for each of Correct, Incorrect, Omission and Percent correct |  | Correct  Learning outcome *F*(1, 20)=4.607, ***p*=.0443**  Session *F*(2.122, 42.44)=78.02, ***p*<.0001**  Interaction *F*(3, 60)=6.336, ***p*=.0008**  Incorrect  Learning outcome *F*(1, 20)=0.03339, *p*=.8569  Session *F*(2.057, 41.14)=14.80, ***p*<.0001**  Interaction *F*(3, 60)=4.216, ***p*=.0090**  Omission  Learning outcome *F*(1, 20)=2.616, *p*=.1215  Session *F*(1.718, 34.35)=11.03, ***p*=.0004**  Interaction *F*(3, 60)=6.571, ***p*=.0006**  Percent correct  Learning outcome *F*(1, 20)=27.42, ***p*<.0001**  Session *F*(2.150, 42.99)=232.0, ***p*<.0001**  Interaction *F*(3, 60)=37.60, ***p*<.0001** | Correct  Last R1: Learned > Did not learn ***p*=.0312**  Omission  Last R1: Learned < Did not learn *p*=.0610  Percent correct  Last R1: Learned > Did not learn ***p*<.0001** |
| **4S5C1** | Two-way ANOVA for each of Correct, Incorrect, Omission and Percent correct | ABA Naïve n=28  ABA Exposed n=22 | Correct  ABA exposure *F*(1, 48)=10.24, ***p*=.0024**  Session *F*(1.506, 72.31)=214.6, ***p*<.0001**  Interaction *F*(3, 144)=2.407, *p*=.0697  Incorrect  ABA exposure *F*(1, 48)=6.251, ***p*=.0159**  Session *F*(2.134, 102.4)=72.12, ***p*<.0001**  Interaction *F*(3, 144)=7.637, ***p*<.0001**  Omission  ABA exposure *F*(1, 48)=6.140, ***p*=.0168**  Session *F*(2.319, 111.3)=25.93, ***p*<.0001**  Interaction *F*(3, 144)=1.722, *p*=.1650  Percent correct  ABA exposure *F*(1, 48)=16.56, ***p*=.0002**  Session *F*(1.635, 78.46)=220.6, ***p*<.0001**  Interaction *F*(3, 144)=6.042, ***p*=.0007** | Correct  First PD: ABA Naïve > ABA Exposed ***p*=.0001**  Incorrect  First PD: ABA Naïve > ABA Exposed ***p*=.0005**  Omission  First PD: ABA Naïve < ABA Exposed *p*=.0537  Percent correct  First PD: ABA Naïve > ABA Exposed ***p*<.0001** |
| **4S5C2** | Two-way ANOVA for each of Correct, Incorrect, Omission and Percent correct |  | Correct  ABA exposure *F*(1, 48)=15.30, ***p*=.0003**  Session *F*(2.150, 103.2)=132.5, ***p*<.0001**  Interaction *F*(3, 144)=9.290, ***p*<.0001**  Incorrect  ABA exposure *F*(1, 48)=21.22, ***p*<.0001**  Session *F*(1.704, 81.82)=60.71, ***p*<.0001**  Interaction *F*(3, 144)=12.19, ***p*<.0001**  Omission  ABA exposure *F*(1, 48)=0.7449, *p*=.3924  Session *F*(2.341, 112.4)=30.27, ***p*<.0001**  Interaction *F*(3, 144)=1.979, *p*=.1198  Percent correct  ABA exposure *F*(1, 48)=16.52, ***p*=.0002**  Session *F*(1.585, 76.08)=228.4, ***p*<.0001**  Interaction *F*(3, 144)=6.971, ***p*=.0002** | Correct  First PD: ABA Naïve > ABA Exposed ***p*<.0001**  Incorrect  First PD: ABA Naïve > ABA Exposed ***p*<.0001**  First R1: ABA Naïve > ABA Exposed ***p*=.0024**  Percent correct  First PD: ABA Naïve > ABA Exposed ***p*<.0001** |
