# Supplementary material for: Rapid, automated, and experimenter-free touchscreen testing reveals reciprocal interactions between cognitive flexibility and activity-based anorexia in female rats
Source: eLife. 2023 Jun 30;12:e84961. doi: 10.7554/eLife.84961 (PMC10344425; doi:10.7554/eLife.84961)
Supplement: Figure 5—source data 1. [file elife-84961-fig5-data1.docx]

**Figure 5 Statistics**

| **Figure** | **Statistical test** | **Group n** | **Main analysis result** | **Significant post-hoc multiple comparisons** |
| --- | --- | --- | --- | --- |
| **5B** | Log-rank (Mantel-Cox) test | After Reversal Task (Susceptible n=11, Resistant n=14)  Before Reversal Task (Susceptible n=21, Resistant n=1) | χ^2^(1)=16.88, ***p*<.0001** |  |
| **5D** | Unpaired t test |  | *t*(45)=4.855, ***p*<.0001** |  |
| **5E** | Unpaired t test |  | *t*(45)=3.557, ***p*=.0009** |  |
| **5F** | Baseline: Mixed-effects analysis | After Reversal Task (Susceptible n=10, Resistant n=13)  Before Reversal Task (Susceptible n=21, Resistant n=1) | Time *F*(6, 245)=44.0, ***p*<.0001**  ABA timing *F*(1, 43)=28.5, ***p*<.0001**  Interaction *F*(6, 245)=10.4, ***p*<.0001** | Day 3: Before Reversal Task > After Reversal Task ***p*=.0440**  Day 4: Before Reversal Task > After Reversal Task ***p*=.0105**  Days 5-7: Before Reversal Task > After Reversal Task all ***p*s<.0001** |
| **5G** | Two-way RM ANOVA |  | Phase *F*(1, 43)=278.9, ***p*<.0001**  ABA timing *F*(1, 43)=16.5, ***p*=.0002**  Interaction *F*(1, 43)=2.556, *p*=.1172 | Baseline: Before Reversal Task > After Reversal Task ***p*=.0160**  ABA: Before Reversal Task > After Reversal Task ***p*<.0001** |
| **5H** | Two-way RM ANOVA |  | Phase *F*(1, 43)=31.18, ***p*<.0001**  ABA timing *F*(1, 43)=19.93, ***p*<.0001**  Interaction *F*(1, 43)=0.5208, *p*=.4744 | Baseline: After Reversal Task > Before Reversal Task ***p*=.0010**  ABA: After Reversal Task > Before Reversal Task ***p*<.0001** |
| **5I** | Unpaired t test  Only includes ABA Susceptible animals | After Reversal Task Susceptible n=11  Before Reversal Task Susceptible n=21 | *t*(30)=1.566, *p*=.1277 |  |
| **5J** | Unpaired t test  Only includes ABA Susceptible animals |  | *t*(30)=0.2563, *p*=.7994 |  |
| **5K** | Baseline: Mixed-effects analysis  Only includes ABA Susceptible animals | After Reversal Task Susceptible n=10  Before Reversal Task Susceptible n=21 | Time *F*(6, 169)=22.7, ***p*<.0001**  ABA timing *F*(1, 29)=17.6, ***p*=.0002**  Interaction *F*(6, 169)=5.22, ***p*<.0001** | Day 5: Before Reversal Task > After Reversal Task ***p*=.0001**  Days 6-7: Before Reversal Task > After Reversal Task all ***p*s<.0001** |
| **5L** | Two-way RM ANOVA  Only includes ABA Susceptible animals |  | Phase *F*(1, 29)=225.7, ***p*<.0001**  ABA timing *F*(1, 29)=8.583, ***p*=.0065**  Interaction *F*(1, 29)=0.1r410, *p*=.7100 | Baseline: Before Reversal Task > After Reversal Task ***p*=.0426**  ABA: Before Reversal Task > After Reversal Task ***p*=.0165** |
| **5M** | Two-way RM ANOVA  Only includes ABA Susceptible animals |  | Phase *F*(1, 29)=32.90, ***p*<.0001**  ABA timing *F*(1, 29)=6.590, ***p*=.0157**  Interaction *F*(1, 29)=0.3683, *p*=.5486 | ABA: After Reversal Task > Before Reversal Task ***p*=.0357** |

**Figure 5-figure supplement 1 Statistics**

| **Figure** | **Statistical test** | **Group n** | **Main analysis result** | **Significant post-hoc multiple comparisons** |
| --- | --- | --- | --- | --- |
| **5S1B** | Unpaired t test | Before Reversal Task (Susceptible n=21, Resistant n=1)  Food restriction only n=22 | *t*(42)=10.77, ***p*<.0001** |  |
| **5S1C** | Unpaired t test |  | *t*(42)=3.004, ***p*=.0045** |  |
