# Supplementary material for: Rapid, automated, and experimenter-free touchscreen testing reveals reciprocal interactions between cognitive flexibility and activity-based anorexia in female rats
Source: eLife. 2023 Jun 30;12:e84961. doi: 10.7554/eLife.84961 (PMC10344425; doi:10.7554/eLife.84961)
Supplement: Supplementary file 1. — Parameters for pretraining and 2VDLR in the novel touchscreen apparatus. [file elife-84961-supp1.docx]

**Table S3 Touchscreen Parameters.** Parameters for pretraining and 2VDLR in the novel touchscreen apparatus

| **Stages** | **Response** | **Trial Outcome** | **Reward** | **Trial Initiation** | **Progression Criterion** |
| --- | --- | --- | --- | --- | --- |
| **Habituation** | N/A | N/A | Sucrose pellets spread throughout PhenoSys | N/A | Consumption of 15-20 pellets in testing chamber |
| **Initial Touch** | Touch illuminated square | Correct tone + magazine LED illumination | 2 sucrose pellets | Automatic start after 10s ITI end | 2 sessions with 30 (maximum) trials |
|  | Touch to blank window during stimulus presentation |  | 1 sucrose pellet |  |  |
|  | No screen touch |  |  |  |  |
| **Must Touch** | Touch illuminated square | Correct tone + magazine LED illumination | 1 sucrose pellets | Automatic start after 10s ITI end | 2 sessions with 30 (maximum) trials |
|  | Touch to blank window during stimulus presentation | No effect; trial continues until stimulus is touched | | |  |
| **Must Initiate** | Touch illuminated square | Correct tone + magazine LED illumination | 1 sucrose pellets | Must be initiated by magazine nose poke after 10s ITI end | 2 sessions with 30 (maximum) trials |
|  | Touch to blank window during stimulus presentation | No effect; trial continues until stimulus is touched | | |  |
| **Punish Incorrect** | Touch illuminated square | Correct tone + magazine LED illumination | 1 sucrose pellets | Must be initiated by magazine nose poke after 10s ITI end | 2 sessions with 30 (maximum) trials with ≥80% accuracy |
|  | Touch to blank window during stimulus presentation | Incorrect tone + house light on + 5s time out | No reward | Must be initiated by magazine nose poke after 5s time out + 15s ITI end |  |
|  | No screen touch |  |  |  |  |
| **Pairwise Discrimination + Reversal Learning** | Touch to correct image | Correct tone + magazine LED illumination | 1 sucrose pellets | Must be initiated by magazine nose poke after 10s time out + 10s ITI end | 2 sessions with 30 (maximum) trials with ≥80% accuracy |
|  | Touch to incorrect image | Incorrect tone + house light on + 10s time out | No reward |  |  |
|  | No screen touch |  |  |  |  |

**ITI:** inter-trial interval.
